# 110 Seven-Year Analysis of National Rehabilitation Trends and Outcomes Among Burn Patients

**DOI:** 10.1093/jbcr/irae036.109

**Published:** 2024-04-17

**Authors:** Darby Little, Stephanie A Mason

**Affiliations:** University of Toronto, Toronto, ON; Sunnybrook Health Sciences Centre, Toronto, ON; University of Toronto, Toronto, ON; Sunnybrook Health Sciences Centre, Toronto, ON

## Abstract

**Introduction:**

The recovery journey for individuals with major burns is often protracted, necessitating extended stays at rehabilitation facilities before they can return home. We aimed to describe national resource utilization and outcomes related to inpatient rehabilitation following burn injury.

**Methods:**

In this retrospective population-based study, we examined all admissions to inpatient rehabilitation facilities from April 1, 2016-March 31, 2023, available from a national repository of health administrative data. This database aggregates information from participating adult inpatient rehabilitation facilities (including 104 programs by May 2022) in 9 provinces.

**Results:**

During the 7-year study period, we identified 513 burn patients who were admitted to inpatient rehabilitation in the database. Burn patients were overall younger, with a greater proportion of burn survivors under 45 years of age (25.9%-54.2%) compared to the general rehabilitation population (5.5%-6.1%). Throughout the study period, burn patients consistently experienced a longer median length of stay, which generally increased from 26.5 days in 2017 to 30.5 days in 2023. In contrast, the median length of stay for the general rehabilitation population remained stable between 22.0-23.0 days. Throughout the study period, 66.1% of burn patients reported improved pain levels upon discharge from the rehabilitation facility. Moreover, burn patients demonstrated enhancements in their motor functional scores, with an average increase score of 20.3-29.8 per year, as determined by the 18-item Functional Independence Measure instrument.

**Conclusions:**

Burn patients experience extended rehabilitation admissions when compared to the general rehabilitation population. Their improvements in pain and functional scores emphasize the importance of rehabilitation in facilitating their recovery and improving their quality of life.

**Applicability of Research to Practice:**

This national population-based study underscores the importance of rehabilitation services in the recovery of burn patients.

